# Success in Medicine, and the Way It May Be Achieved

**Published:** 1904-10

**Authors:** 


### SUCCESS IN MEDICINE, AND THE WAY IT MAY BE ACHIEVED.*

As the subject of my remarks I have selected "Success in Medicine, and the Way It May Be Achieved," thinking it would be of interest to our young graduates who are about to be committed to the tender mercies of a more or less confiding public.

It is a well-known fact that not all who receive the degree of Doctor of Medicine make a success of the practice of medicine. Some become impatient with the long time it may be necessay to wait for patients; others tire of the hardships inseparable from the calling—the physical wear and tear, the mental strain and responsibility; while still others, not having the love of the profession in their hearts, decide to try a shorter and easier way to fame or fortune.

It has been estimated that something like ten per cent. of physicians fall out of the ranks within ten years in order to engage in other pursuits. Of my own class of twenty-one, graduated twenty-five years ago, only one has voluntarily given up his profession, and he still keeps in touch with it—having become a medical drummer; six entered the United States medical services, and four have answered the last "sick call" and have joined the great majority.

*Address by George Tully Vaughan, M.D., Washington, D. C., before the graduates of the Medical School of Georgetown University, June 7, 1904.

Success in medicine means something more than earning a living, gaining wealth, or making a great reputation—it means *doing things* to improve the general welfare—to add to the sum of human happiness; and I believe that every physician worthy of his calling, and all great physicians, have been actuated by such desires. If your object is to amass wealth, choose some other line of business—you are on the wrong road. It is true that affluence and even wealth are not inconsistent with the proper practice of your profession, but while these are exceptional, there is nearly always for the doctor who attends to his business, not only a good living, but the confidence and affection of the community. One of the attributes most essential to success is *sympathy*—not a maudlin sentiment, but true appreciation and pity for and desire to relieve the patient's trouble and suffering. It is the quality, beyond all others, which ennobles the profession.

Having selected a suitable location or undertaken any particular line of work, be *persistent*—don't be discouraged. Stick to it, and you will succeed. I once knew a young doctor who did not make enough during his first year of practice to pay one month's board, but in less than three years, by close attention to business, he had the patronage of almost the entire community. Don't be discouraged if your first patients fail to respond to treatment and, in spite of your best efforts, insist on dying. A celebrated surgeon was so discouraged at the beginning of his career because his first two patients died, that he pulled down his sign, threw it into an old well, and decided to abandon the profession. Happily for the benefit of mankind, circumstances prevented the carrying out of his hasty resolution, and he lived to accomplish a work which has afforded relief to a class of sufferers who, previous to his day, had been doomed to lives of hopeless misery.

*Be honest and truthful*—at the same time be *tactful*—but do not be brusque in your honesty or deceitful in your tact. Unpleasant information can be communicated in a gentle, sympathetic way and at the same time with perfect truth and sincerity, so that the blow is softened, the sting of disappointment or of grief is diminished. On the other hand, ordinary advice may be communicated in such a tactless or even boorish manner that offence is given. Tact means touch. Tactful is knowing how to touch your patients—not necessarily in their pocket-books, although this is not to be neglected—but in their feelings and sentiments.

It is the faculty of doing nicely whatever is best under the circumstances—a faculty which you can not afford to neglect—at least not while you are young, struggling, impecunious doctors. When you have grown famous, rich and independent, you may affect abrupt and boorish manners.

Many stories might be told of the disastrous results of the practice of honesty and truth untempered with tact. I will select only one. The young physician says: "The case was that of a woman whom I considered distinctly old, although I now realize that fifty-five is not hopelessly venerable. She had always been very active and was annoyed to find herself losing strength and becoming easily exhausted. 'I have always done these things; why can't I now?' she demanded. And I, out of my guileless youth, explained, 'But you can't expect to—at your age.' Naturally that settled me. She sent for an old fossil of seventy, who had not half of my actual learning, but who could say the right things, and so still held a thriving practice."

*Morality.*—It goes without saying that one who holds the delicate relations which often exist between physician and patient must be of good character. A distinguished professor of surgery, in his address to a graduating class, advised them to get married as soon as they could, as it would thus be a guaranty to the public of good behavior, an evidence that the days of sowing wild oats were passed—and it would inspire confidence in their friends and add to your own dignity. I believe that this is good advice, and would only caution you not to talk *too* much to your wives. There are some things you had better not tell them.

The confessions made by the patient to the physician must be held sacred by the latter and shared by him with no one. A well-known English physician, Dr. Playfair, told his wife a professional secret; she told her most intimate friend. The result was that the doctor, after a law suit, was compelled to pay the sum of $60,000 to the indignant patient.

*Courage and Self-Denial.*—In the days before the discovery of anesthesia it required courage to become a surgeon. The one with the ordinary feelings of humanity, the evident suffering of the patient under operation, whether shown by compressed lips and set teeth, or by shrieks and groans, must have been a fearful strain on the surgeon. There are still many occasions not only on the battle-field, in epidemics of disease, in self-imposed experiments, but in the every-day life of the physician which require the highest order of courage—especially moral courage—courage to do right and to refuse to do wrong. Many times the temptation may require all your strength and self-denial to resist, and it may come in such guise as almost to convince you that to yield would be right.

Let no unworthy desire for wealth inveigle you into questionable practice. If there is any question about the propriety of an act in contemplation, give your character the benefit of the doubt and don't do it. Have the manliness to shut out jealousy; or if you must admit its existence in your own breast—don't tell it, don't do anything to make any one suspect it—keep it a dead secret within yourself. It has been said that the medical profession has more jealousy among its members than any other profession. That ought not to be among those who are actuated by the true spirit of their profession, and it usually means too little philanthropy and too much commercialism.

In an article by Mr. Goodrich, he divides medical men into professional doctors, merchant doctors, and quacks, and remarks that it is the professional doctor—the true physician, working regardless of fees—that holds the profession to its philanthropic ideal.

*Versatility.*—The physician should be a man of versatile accomplishments—a many-sided man; he should know something about everything and everything about his particular line of specialty. Our forefathers seem to have appreciated versatility in their medical men, as is shown by the following clipping by Conan Doyle from a newspaper of 1787:

"Wanted—For a family not blessed with good health, a sober, discreet and steady person to act in the capacity of doctor and apothecary. He must often act also as a steward and butler, and occasionally dress hair and wigs. He will be required to read prayers, and sometimes, on wet Sundays, to preach a sermon or two. A good salary will be paid, and a preference will be given to such an one as, besides the above qualifications, can mend clothes."

*Prototypes.*—As man is in the highest degree an imitative animal, it is well on the threshhold of a career to select examples from the histories of great men whose lives have been characterized by some particular virtue worthy of emulation. For *persistence,* take Marion Sims, who, refusing to be discouraged by twenty-nine failures, performed the thirtieth operation—all on the same patient—and succeeded, opening thereby the door of hope to a class of patients previously considered incurable.

For *courage,* take Ephraim McDowell, that pioneer in surgery, who dared, for the relief of his patient, to perform an operation unheard of before—when he knew that failure would have meant for him indictment and trial for murder.

For *simple faith,* but at the same time faith not without works, take the old French army surgeon, Ambrose Pare. In the case of Count Mansfield, who was seriously wounded at the battle of Moncontour, when Pare first examined the wound he thought it incurable; but after prolonged treatment and ultimate recovery, he refers to him "whom I dressed and whom God healed." And again, in the case of Marquis d'Auret. In the consultation when the other surgeons pronounced it a hopeless case, Pare says: "I told them there was still some hope, because he was young, and God and nature sometimes do things which seem to physicians and surgeons impossible." This proved to be another case where Pare dressed the patient and God healed him.

As a type of *industry and energy* in the pursuit of knowledge, take John Hunter, who for a good part of his life was in the habit of working nineteen hours out of the twenty-four, and did more to advance the science of surgery than any man who lived in the days before anesthesia had been discovered. Industry—love of work—without genius is better than genius without industry. It is an interesting question as to which is more important in determining success—opportunity or industry—both are important. Shakespeare says: "There is a tide in the affairs of men which, taken at the flood, leads on to fortune." But without industry you may fail to seize your opportunity and get the advantage of the flood. Every man—and every woman, too—is given at least one chance for success.

According to Ingalls, opportunity knocks unbidden once at every gate:

> "Master of human destinies am I.
> Fame, love and fortune on my foot wait,
> Cities and fields I walk—I penetrate
> Deserts and seas remote, and passing by
> Hovel and mart and palace—soon or late
> I knock unbidden once at every gate.
> If sleeping, wake—if feasting, rise before
> I turn away. It is the hour of fate,
> And they who follow me reach every state
> Mortals desire, and conquer every foe
> Save death; but those who doubt or hesitate,
> Condemned to failure, penury and woe,
> Seek me in vain and uselessly implore,
> I answer not, and I return no more."

In the Hall of Fame—the American Valhalla—where the names of our most distinguished Americans are supposed to go when their bodies die, the names of many lawyers, soldiers and business men, but remarkable to relate, not one physician. This may be regarded as a tribute to the modesty of the physician—his work is done so quietly and unostentatiously that few of the public realize that it is worthy of recognition in the shape of monuments and memorials; but in closing, I would like to ask: Is the *lawyer* or jurist, who protects society by making and enforcing a good system of laws, a greater benefactor than the *physician*, who protects society by giving it a remedy which prevents smallpox and thus saves millions from sickness and death? Is the *business man*, who amasses great wealth, though he may spend it generously, give employment to many and endow colleges and charitable institutions, more deserving of gratitude than the *physician*, who introduces a remedy which annihilates pain and permits the performance of the most frightful operations while the patient sleeps in blissful unconsciousness?

Is the *soldier*, who changes forms of governments and the map of nations, perhaps sometimes for the better, but at the cost of incalculable treasure and the suffering and death of myriads, more worthy of honor and fame than the *physician*, who saves millions of dollars to commerce and untold numbers of human beings from sickness and death by giving to the world a method for the management and prevention of yellow fever?—*Virginia Medical Semi-Monthly*.

